# Global, regional, and national burden of cataract among older adults from 1990 to 2021: a comprehensive analysis based on the global burden of disease study 2021

**DOI:** 10.3389/fmed.2025.1679828

**Published:** 2025-09-04

**Authors:** Zhongqi Wan, Jianhao Bai, Weifang Wang, Qing Peng

**Affiliations:** ^1^Department of Ophthalmology, Shanghai Tenth People's Hospital Affiliated to Tongji University, Tongji University School of Medicine, Shanghai, China; ^2^Department of Ophthalmology, Shanghai East Hospital Affiliated to Tongji University, Tongji University School of Medicine, Shanghai, China; ^3^Department of Ophthalmology, Shanghai Eighth People's Hospital, Shanghai, China

**Keywords:** cataract, older adults, global burden of disease, age-standardized prevalence, health inequality

## Abstract

**Objective:**

To assess the global, regional, and national burden of cataract among individuals aged 60 years and older from 1990 to 2021, and to examine disparities by age, sex, socio-demographic level, and geographic region using data from the Global Burden of Disease Study (GBD) 2021.

**Methods:**

We extracted cataract prevalence data for 204 countries from GBD 2021 and analyzed age-standardized prevalence rates (ASPRs) across regions and Socio-demographic Index (SDI) levels. Sex- and age-specific patterns were assessed. Age-period-cohort modeling, decomposition analysis, absolute and relative inequality metrics, and frontier analysis were applied to assess temporal trends, demographic drivers, and disparities.

**Results:**

In 2021, the global ASPR of cataract among the elderly was 7,748.5 per 100,000, with significantly higher rates in South Asia. Females had consistently higher ASPRs than males across all age groups and regions. Prevalence increased with age, peaking in those aged ≥95 years. Age-period-cohort (APC) analysis revealed that aging is the dominant driver of burden, with minor period and cohort effects. Decomposition showed that global prevalence increases were largely driven by population growth (87.4%), with smaller contributions from aging and epidemiological change. Substantial inequality persisted: low-SDI countries bore disproportionately higher burdens, with minimal improvement from 1990 to 2021. Frontier analysis revealed large performance gaps even among similarly developed countries.

**Conclusion:**

Cataract remains a major and unequal public health burden among older adults, particularly in low- and middle-SDI settings. Addressing service delivery inefficiencies, expanding surgical coverage, and implementing equitable aging-focused eye care policies are essential to reduce avoidable visual impairment globally.

## Introduction

Cataract remains the predominant cause of reversible blindness globally, responsible for over 30% of all instances of visual impairment and blindness, as indicated by recent global estimates (1, 2). Cataract is characterized by the progressive opacification of the eye's crystalline lens and disproportionately affects older adults, with both incidence and severity increasing significantly after the age of 60 (3). Consequently, the burden of cataract is closely associated with population aging and poses a substantial challenge to achieving healthy longevity. Beyond its direct impact on visual acuity, cataract is linked to a wide array of adverse health outcomes (4, 5). Visual impairment from cataract is associated with reduced quality of life and impaired mobility. It also increases the risk of falls, fractures, and accelerated cognitive decline in older individuals (6, 7). These functional limitations lead to increased healthcare utilization, loss of independence, and psychosocial distress, thereby exacerbating the societal and economic burden of the disease.

The global population aged 60 years and older is projected to more than double by 2050 (8). As a result, age-related eye conditions such as cataracts are expected to become increasingly important public health challenges (9). This demographic shift will not only increase the absolute number of individuals affected but also place additional strain on healthcare systems, particularly in regions where access to surgical interventions and eye care infrastructure remains inadequate. It is therefore, imperative to comprehend the evolving patterns and determinants of the cataract burden in older populations to inform effective, equitable, and forward-looking policy responses.

Although cataracts are widely acknowledged as a leading cause of visual impairment, most existing studies focus on single countries or regions, such as China, India, or parts of Sub-Saharan Africa (10–12). These studies primarily investigate local prevalence or surgical coverage. However, these studies often lack a comprehensive global or longitudinal perspective (13, 14), and few have systematically analyzed the evolving burden among older adults, who are disproportionately affected (15, 16). Furthermore, there has been insufficient attention given to the interaction between epidemiological trends and key structural determinants, such as population aging, health system capacity, gender disparities, and socio-demographic development. Cross-national comparisons and forward-looking projections, which are crucial for effective health planning and policy formulation, remain inadequately explored. Although the Global Burden of Disease (GBD) framework provides standardized global estimates, there is a notable lack of focused analyses specifically targeting the elderly population (aged 60 and above) that incorporate multi-dimensional drivers of inequality (17–20). Most previous global or regional studies have either pooled all age groups, relied primarily on descriptive temporal trends, or applied a single analytical method (21, 22). Such approaches limit the ability to disentangle demographic from epidemiologic drivers, to evaluate equity dimensions, and to inform targeted interventions for older adults.

This study seeks to address these critical gaps by offering a comprehensive and age-specific assessment of the global, regional, and national burden of cataract among individuals aged 60 years and older from 1990 to 2021, utilizing data from the GBD Study 2021. Through the analysis of age-standardized prevalence across 204 countries and the stratification of trends by age, sex, region, and socio-demographic development level, this study provides detailed insights into the distribution and inequality of cataract burden in the elderly population. In addition, we employed age-period-cohort (APC) modeling, decomposition analysis, inequality indices, and frontier benchmarking to examine temporal dynamics, quantify systemic disparities, and identify key structural drivers shaping the global burden.

## Materials and methods

### Data source and study population

Data for this study were obtained from the GBD Study 2021 (GBD 2021), a comprehensive and standardized effort led by the Institute for Health Metrics and Evaluation to estimate the global burden of diseases, injuries, and risk factors. We extracted estimates for cataract prevalence from the Global Health Data Exchange (GHDx, https://ghdx.healthdata.org), specifically focusing on individuals aged 60 years and older. The analysis covered 204 countries and territories across 21 GBD-defined regions from 1990 to 2021. The primary outcome was the age-standardized prevalence rate (ASPR) per 100,000 population, accompanied by 95% uncertainty intervals (UIs).

### Case definition and disease modeling

Cataract was defined according to the GBD cause list and mapped to the International Classification of Diseases, Tenth Revision (ICD−10) codes H25–H26. Prevalence estimates were modeled using DisMod-MR 2.1, a Bayesian meta-regression tool that synthesizes diverse data sources, including population-based surveys, administrative records, hospital data, and published literature (23). Age standardization was performed using the GBD global standard population. All estimates are presented with 95% UIs derived from 1,000 posterior draws to reflect uncertainty. In countries with limited or poor-quality primary data, DisMod-MR 2.1 borrows information from epidemiologically similar countries within the same GBD region, incorporates predictive covariates, and applies Bayesian hierarchical modeling to generate estimates. The uncertainty arising from sparse data is propagated through posterior sampling, leading to wider 95% uncertainty intervals in such settings.

### Statistical analysis

We analyzed age-standardized prevalence rates (ASPRs) at the global, regional, and national levels and stratified countries into five groups based on the Socio-demographic Index (SDI): low, low-middle, middle, high-middle, and high (24). Sex- and age-specific analyses were conducted across 5-year age groups from 60–64 years to ≥95 years. Absolute and relative differences by sex and SDI level were assessed. All GBD-based statistical analyses were consistent with GBD 2021 methodology.

In addition, all data were processed and analyzed using Microsoft Excel 2021 and R software version 4.3.3. After data extraction, preprocessing and cleaning were conducted, followed by statistical analysis and visualization using the dplyr, ggplot2, and officer packages in R. A two-sided *P* value < 0.05 was considered statistically significant unless otherwise specified.

To explore temporal dynamics, we performed APC analysis using standard log-linear models, estimating net drift, local drift, and adjusted age, period, and cohort effects. Decomposition analysis was applied to quantify the relative contributions of population growth, population aging, and epidemiological change to the overall change in cataract burden between 1990 and 2021, using the Das Gupta method.

For inequality assessment, we calculated the Slope Index of Inequality (SII) and the Concentration Index (CII) to quantify absolute and relative disparities in ASPR across the SDI spectrum. Finally, frontier analysis was conducted by constructing a non-parametric SDI-ASPR efficiency frontier to evaluate national-level deviations from the theoretical minimum burden. All advanced analyses and visualizations were implemented in R and Stata.

## Results

### Geographic distribution of age-standardized prevalence of cataract among the elderly in 2021 and future projections to 2050

**Figure 1:**
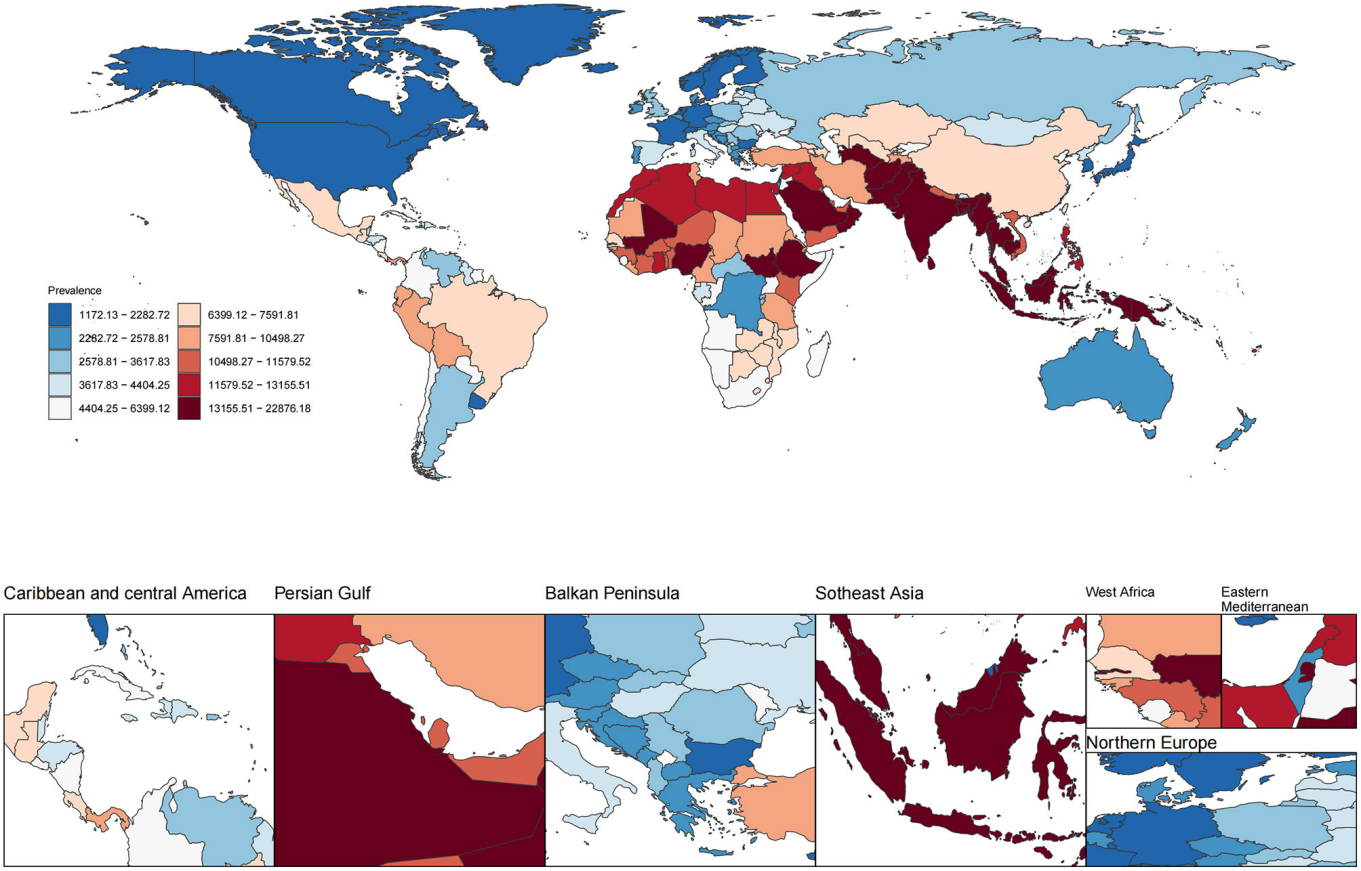
Global distribution of age-standardized prevalence rate (ASPR) of ocular tumors in 2021. The choropleth map illustrates the age-standardized prevalence rate (per 100,000 population) of ocular tumors across 204 countries and territories in 2021. Color gradients represent deciles of ASPR.

In 2021, the global ASPR of cataract among individuals aged 60 years and older was 7,748.5 (UI 6,285.7–9,569.7) per 100,000 population (Figure 1, Table 1). Marked geographic differences in ASPR were observed across regions and socio-demographic development levels. The highest ASPRs were reported in South Asia (17,756.6, UI 14,672.8–21,468.7), Oceania (17,002.6, UI 13,754.9–20,916.3), Western Sub–Saharan Africa (14,418.6, UI 11,900.4–17,474.8), and Southeast Asia (14,005.9, UI 11,982.2–16,321.5). Several other regions, including North Africa and the Middle East (10,878.9, UI 8,674.3–13,524.0) and Eastern Sub-Saharan Africa (9,441.6, UI 7,811.6–11,350.4), also showed rates exceeding 9,000 per 100,000. By contrast, the lowest ASPRs were observed in High-income Asia Pacific (1,810.8, UI 1,372.1–2,372.3), Australasia (2,329.7, UI 1,742.0–3,062.6), High SDI regions (2,363.2, UI 1,803.5–3,072.6), and Western Europe (2,774.1, UI 2,108.0–3,600.2). Other regions with relatively low rates included Central Europe (2,676.8, UI 1,965.2–3,583.8), Eastern Europe (3,555.0, UI 2,670.1–4,661.6), and Southern Latin America (3,697.8, UI 2,769.5–4,824.6). When stratified by SDI level, the low-middle SDI group had the highest ASPR at 15,324.9 (UI 12,685.5–18,504.6), followed by low SDI (12,909.4, UI 10,675.0–15,617.4) and middle SDI (9,612.9, UI 7,868.1–11,779.6). In contrast, high-middle SDI and high SDI groups reported notably lower rates of 5,931.0 (UI 4,678.5–7,482.4) and 2,363.2 (UI 1,803.5–3,072.6), respectively. These results reveal substantial heterogeneity in the age-standardized prevalence of cataract among older adults across regions and development levels in 2021.

**Table 1:** Global and regional ASPR of cataract among adults aged ≥60 years in 1990 and 2021, with 95% uncertainty intervals.

**Location name**	**1990 (ASPR per 100,000, 95% UI)**			**2021 (ASPR per 100,000, 95% UI)**		
	**Both sexes**	**Male**	**Female**	**Both sexes**	**Male**	**Female**
Global	7,399.30 (6,082.64, 8,999.77)	7,164.50 (5,887.56, 8,712.28)	7,607.50 (6,251.26, 9,250.51)	7,748.52 (6,285.66, 9,569.68)	7,080.37 (5,713.72, 8,788.17)	8,300.27 (6,750.81, 10,216.17)
East Asia	6,091.59 (4,916.62, 7,533.26)	5,247.49 (4,197.83, 6,555.30)	6,684.17 (5,409.68, 8,230.50)	6,851.94 (5,431.07, 8,625.74)	5,877.68 (4,611.77, 7,448.31)	7,614.37 (6,045.87, 9,553.42)
Southeast Asia	16,514.11 (13,810.88, 19,673.08)	14,609.28 (12,078.52, 17,546.99)	18,004.76 (15,106.80, 21,336.71)	14,005.93 (11,982.16, 16,321.48)	12,430.91 (10,533.11, 14,585.87)	15,198.66 (13,026.04, 17,647.29)
Oceania	17,765.42 (14,590.71, 21,533.25)	16,218.04 (13,191.00, 19,726.78)	19,316.83 (15,870.97, 23,346.61)	17,002.56 (13,754.90, 20,916.28)	15,837.85 (12,644.91, 19,633.94)	18,208.67 (14,823.04, 22,226.66)
Central Asia	8,189.88 (6,314.43, 10,454.99)	7,450.19 (5,750.77, 9,524.33)	8,572.73 (6,609.63, 10,965.41)	7,637.52 (5,799.48, 9,890.20)	7,046.38 (5,323.25, 9,135.03)	8,013.50 (6,085.31, 10,362.12)
Central Europe	2,777.60 (2,055.91, 3,689.95)	2,518.62 (1,864.26, 3,340.60)	2,931.24 (2,164.40, 3,898.37)	2,676.81 (1,965.23, 3,583.76)	2,448.37 (1,800.50, 3,273.94)	2,818.30 (2,064.61, 3,779.49)
Eastern Europe	3,754.19 (2,850.82, 4,887.76)	3,091.90 (2,345.76, 4,035.64)	3,995.27 (3,031.27, 5,215.45)	3,554.96 (2,670.14, 4,661.60)	2,940.15 (2,205.88, 3,880.16)	3,842.80 (2,881.25, 5,045.65)
High-income Asia Pacific	1,858.48 (1,427.15, 2,399.17)	1,670.76 (1,281.26, 2,161.52)	1,967.02 (1,505.56, 2,542.44)	1,810.81 (1,372.05, 2,372.29)	1,627.71 (1,232.66, 2,129.28)	1,942.60 (1,472.73, 2,548.44)
Australasia	2,370.21 (1,834.46, 3,029.65)	2,173.82 (1,685.07, 2,799.51)	2,482.20 (1,909.90, 3,180.43)	2,329.68 (1,742.02, 3,062.58)	2,150.33 (1,606.62, 2,841.93)	2,473.62 (1,828.80, 3,283.90)
Western Europe	2,910.61 (2,223.59, 3,771.22)	2,411.75 (1,844.53, 3,108.60)	3,182.81 (2,427.48, 4,131.50)	2,774.11 (2,108.04, 3,600.23)	2,312.25 (1,753.84, 3,002.97)	3,109.19 (2,360.03, 4,040.65)
Southern Latin America	3,891.24 (2,957.37, 5,044.14)	3,581.68 (2,706.16, 4,624.94)	4,087.03 (3,104.27, 5,315.95)	3,697.76 (2,769.47, 4,824.56)	3,384.69 (2,530.07, 4,408.65)	3,899.82 (2,913.15, 5,105.48)
High-income North America	1,919.84 (1,478.64, 2,474.53)	1,532.29 (1,183.93, 1,972.96)	2,147.40 (1,647.38, 2,777.20)	1,862.68 (1,432.59, 2,413.42)	1,530.50 (1,175.36, 1,975.64)	2,111.34 (1,617.78, 2,745.04)
Caribbean	4,786.55 (3,662.01, 6,112.37)	4,628.89 (3,515.71, 5,966.95)	4,925.07 (3,792.98, 6,280.66)	4,211.13 (3,164.54, 5,491.29)	4,134.39 (3,091.32, 5,429.39)	4,273.09 (3,214.01, 5,555.53)
Andean Latin America	12,289.36 (9,886.48, 15,083.13)	12,530.26 (10,088.38, 15,378.70)	12,060.01 (9,658.95, 14,835.05)	9,696.29 (7,691.59, 12,093.43)	9,990.47 (7,914.95, 12,449.16)	9,429.17 (7,436.92, 11,845.55)
Central Latin America	7,568.14 (6,076.48, 9,337.33)	7,697.05 (6,221.17, 9,453.03)	7,449.94 (5,926.23, 9,261.82)	6,371.29 (4,992.16, 8,044.63)	6,437.06 (5,069.27, 8,122.92)	6,318.20 (4,928.81, 7,993.92)
Tropical Latin America	7,903.41 (6,458.38, 9,628.72)	7,600.65 (6,181.34, 9,324.35)	8,117.33 (6,649.47, 9,879.24)	6,591.54 (5,271.02, 8,215.49)	6,338.61 (5,044.86, 7,919.16)	6,761.47 (5,419.91, 8,420.39)
North Africa and Middle East	12,214.76 (10,059.40, 14,742.05)	10,860.28 (8,889.84, 13,182.29)	13,558.06 (11,211.71, 16,268.95)	10,878.85 (8,674.29, 13,524.04)	9,759.48 (7,705.40, 12,215.72)	11,984.72 (9,578.89, 14,828.03)
South Asia	21,013.59 (17,739.62, 24,933.45)	18,910.54 (15,866.74, 22,573.61)	23,277.83 (19,683.23, 27,503.98)	17,756.58 (14,672.75, 21,468.69)	16,359.54 (13,414.71, 19,896.81)	19,067.27 (15,812.64, 22,927.43)
Central Sub-Saharan Africa	3,116.47 (2,373.64, 4,058.48)	2,901.55 (2,171.47, 3,831.92)	3,298.30 (2,523.54, 4,261.33)	3,060.05 (2,281.59, 4,051.22)	2,833.61 (2,085.00, 3,768.24)	3,211.92 (2,390.17, 4,281.59)
Eastern Sub-Saharan Africa	10,058.80 (8,435.10, 11,883.34)	9,111.03 (7,601.83, 10,815.86)	10,960.12 (9,224.61, 12,914.94)	9,441.55 (7,811.60, 11,350.43)	8,723.82 (7,174.50, 10,543.86)	10,052.79 (8,332.85, 12,036.99)
Southern Sub-Saharan Africa	7,141.36 (5,932.88, 8,582.52)	6,764.83 (5,584.76, 8,193.40)	7,395.73 (6,165.67, 8,837.48)	6,125.49 (4,990.34, 7,493.19)	5,751.15 (4,645.31, 7,092.55)	6,348.45 (5,182.80, 7,752.29)
Western Sub-Saharan Africa	14,076.25 (11,790.66, 16,837.08)	12,488.53 (10,365.33, 15,072.82)	15,491.52 (13,050.38, 18,407.54)	14,418.57 (11,900.40, 17,474.83)	13,003.21 (10,639.98, 15,891.80)	15,667.40 (13,007.42, 18,899.03)
High-middle SDI	5,128.41 (4,071.42, 6,438.83)	4,629.60 (3,673.26, 5,809.14)	5,419.63 (4,298.36, 6,813.79)	5,931.01 (4,678.49, 7,482.39)	5,297.67 (4,163.73, 6,698.22)	6,381.40 (5,034.64, 8,041.80)
High SDI	2,358.86 (1,813.57, 3,045.15)	1,981.20 (1,528.50, 2,550.05)	2,567.40 (1,967.62, 3,321.18)	2,363.20 (1,803.45, 3,072.61)	2,037.43 (1,557.50, 2,649.67)	2,602.33 (1,984.97, 3,387.39)
Low-middle SDI	17,933.24 (15,131.94, 21,266.84)	16,362.98 (13,725.59, 19,535.99)	19,502.20 (16,467.99, 23,033.40)	15,324.89 (12,685.50, 18,504.55)	14,096.31 (11,583.49, 17,146.85)	16,418.41 (13,613.45, 19,738.72)
Low SDI	13,657.90 (11,484.53, 16,234.48)	12,444.20 (10,379.03, 14,849.40)	14,873.15 (12,584.45, 17,640.40)	12,909.37 (10,675.00, 15,617.44)	11,989.21 (9,863.53, 14,573.83)	13,774.13 (11,439.22, 16,611.42)
Middle SDI	9,983.84 (8,254.81, 12,055.01)	9,137.39 (7,485.83, 11,107.92)	10,678.16 (8,858.32, 12,860.78)	9,612.86 (7,868.09, 11,779.61)	8,730.25 (7,085.49, 10,761.21)	10,345.65 (8,509.23, 12,616.81)

Using Bayesian age–period–cohort modeling, we projected the global burden of cataract among adults aged ≥60 years through 2050 (Supplementary Figure S1). The absolute number of prevalent cases is expected to increase dramatically, reaching 160.8–211.4 million cases by 2050, largely driven by demographic expansion. In contrast, projected ASPRs demonstrated a more moderate upward trend. By 2050, the global ASPR of cataract among the elderly is projected to range between 7,476.9 and 9,832.3 per 100,000.

### Sex-specific age-standardized prevalence of cataract among the elderly in 2021

**Figure 2:**
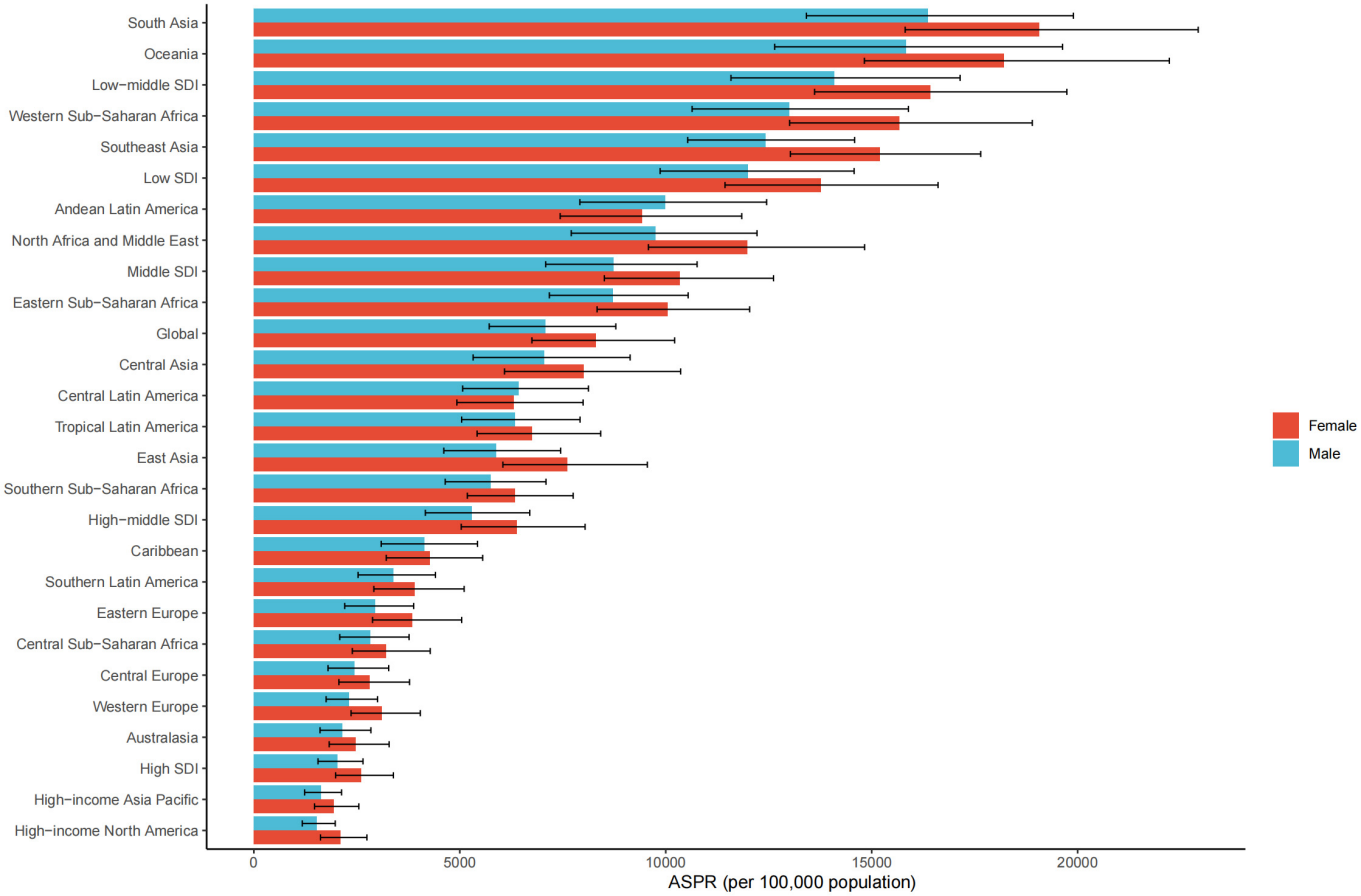
ASPR of ocular tumors by sex, sociodemographic index (SDI) quintile, and GBD region in 2021. Bar plots show ASPR (per 100,000 population) of ocular tumors stratified by sex (male and female), SDI quintile, and 21 Global Burden of Disease (GBD) regions.

In 2021, the global ASPR of cataract was 8,300.3 (UI 6,750.8–10,216.2) per 100,000 among females and 7,080.4 (UI 5,713.7–8,788.2) among males, indicating a higher burden in older women (Figure 2). This sex-related disparity was observed across most regions. The largest absolute differences between females and males were found in South Asia (female: 19,067.3, UI 15,812.6–22,927.4; male: 16,359.5, UI 13,414.7–19,896.8), Western Sub-Saharan Africa (female: 15,667.4, UI 13,007.4–18,899.0; male: 13,003.2, UI 10,640.0–15,891.8), Oceania (female: 18,208.7, UI 14,823.0–22,226.7; male: 15,837.9, UI 12,644.9–19,633.9), and Southeast Asia (female: 15,198.7, UI 13,026.0–17,647.3; male: 12,430.9, UI 10,533.1–14,585.9). In North Africa and the Middle East, the female ASPR was 11,984.7 (UI 9,578.9–14,828.0) compared to 9,759.5 (UI 7,705.4–12,215.7) in males. Sex differences were relatively smaller in high-income regions. For example, in High-income North America, the ASPR was 2,111.3 (UI 1,617.8–2,745.0) in females and 1,530.5 (UI 1,175.4–1,975.6) in males. In High-income Asia Pacific, rates were 1,942.6 (UI 1,472.7–2,548.4) for females and 1,627.7 (UI 1,232.7–2,129.3) for males. A similar pattern was observed in Western Europe, where the female ASPR was 3,109.2 (UI 2,360.0–4,040.7) and the male ASPR was 2,312.3 (UI 1,753.8–3,003.0). By SDI level, the largest sex gap was found in low-middle SDI regions (female: 16,418.4, UI 13,613.5–19,738.7; male: 14,096.3, UI 11,583.5–17,146.9), followed by low SDI (female: 13,774.1, UI 11,439.2–16,611.4; male: 11,989.2, UI 9,863.5–14,573.8). In high SDI settings, the sex difference was narrower, with ASPRs of 2,602.3 (UI 1,985.0–3,387.4) in females and 2,037.4 (UI 1,557.5–2,649.7) in males.

**Figure 3:**
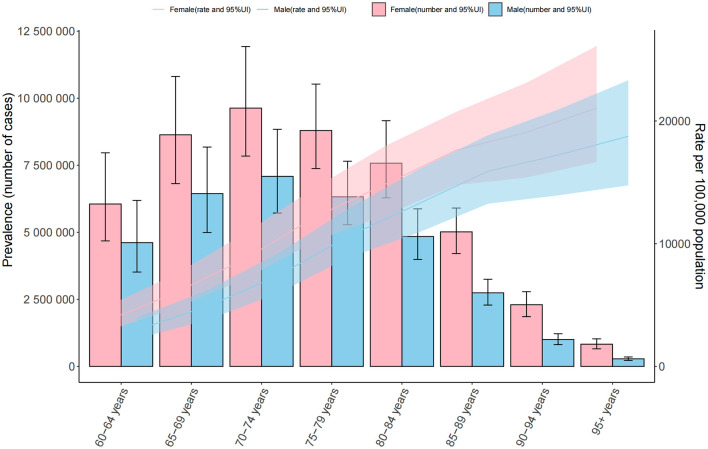
Age-specific number and rate of ocular tumor prevalence in 2021 by sex. The dual-axis chart displays age-specific prevalence of ocular tumors across age groups from 60–64 to 95+ years in 2021. Bars represent the number of prevalent cases (left *Y*-axis), and lines represent ASPR per 100,000 population (right *Y*-axis), with 95% UIs. Male and female data are distinguished by color and line type.

Further analysis of age-specific patterns showed that the age-standardized prevalence of cataract increased consistently with age in both sexes. Among males, the rate rose from 2,966.6 per 100,000 population in the 60–64 years age group to 18,753.8 in those aged 95 years and above. Among females, the corresponding rates were higher in each age group, ranging from 3,681.7 in the 60–64 group to 21,049.3 in the 95+ age group. In terms of absolute numbers, the highest burden was observed in the 70–74 years group, with 7.1 million male and 9.6 million female prevalent cases, respectively. Although the number of cases declined in older age groups due to population reduction, the prevalence rates continued to rise, peaking in the oldest group (≥95 years). Across all age intervals, females consistently exhibited higher prevalence rates and case numbers than males, with the sex gap widening with advancing age. This pattern indicates that the burden of cataract disproportionately affects elderly women, particularly in the very old age strata (Figure 3).

### Temporal and generational trends in cataract prevalence based on age-period-cohort analysis

**Figure 4:**
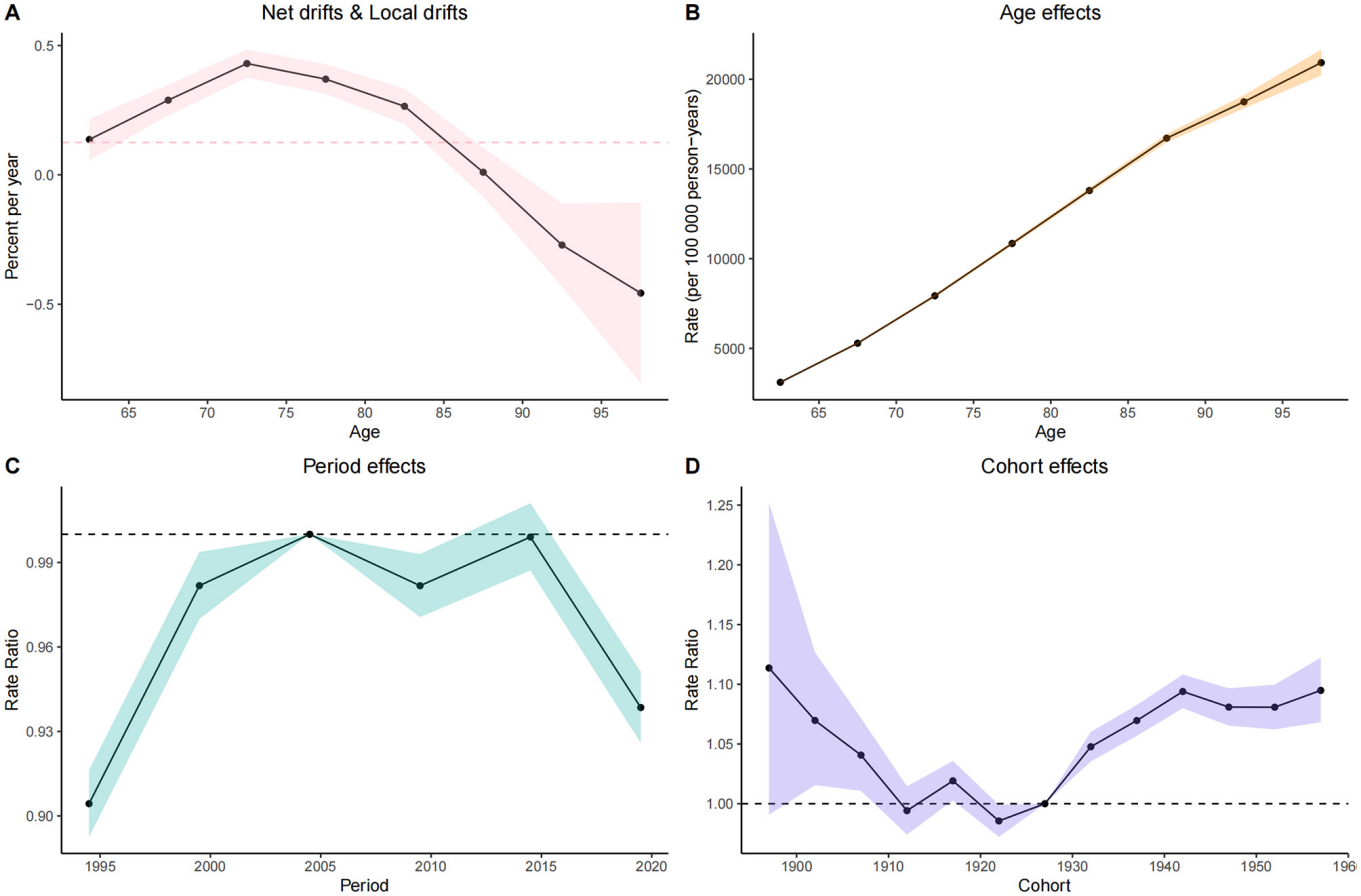
Age-Period-Cohort (APC) analysis of ocular tumor prevalence. The panels display components of the APC model. **(A)** Local and net drift curves depict age-specific and overall temporal trends in prevalence. **(B)** Age effects show modeled prevalence rates across age groups (65–95+). **(C)** Period effects reveal relative changes over calendar years (1990–2020). **(D)** Cohort effects demonstrate the influence of birth cohorts (1900–1960) on disease burden. All estimates are adjusted for age, period, and cohort effects and reflect changes in log prevalence rate ratios.

APC analysis was conducted to evaluate dynamic changes in cataract prevalence over time and across age groups and birth cohorts. In the net drift and local drift analysis (Figure 4A), the overall net drift was slightly above 0%, indicating a modest annual increase in prevalence across the observation period. The local drift curve revealed age-specific annual percent changes, with the highest drift observed in the 70–74 age group, followed by a gradual decline in older age groups. This suggests that the greatest temporal increase in prevalence occurred around early elderly stages, whereas the rate of increase diminished among the oldest-old. The age effect curve (Figure 4B), adjusted for period and cohort influences, showed a continuous increase in prevalence with advancing age, peaking near 90 years. This confirmed aging as an independent and dominant factor in cataract burden.

The period effect (Figure 4C) displayed a non-linear trend. From 1995 to 2005, the rate ratio increased, indicating a rising burden during this time. However, the upward trend slowed after 2000, and prevalence began to decline between 2005–2010 and 2015–2020. These turning points coincided with broader adoption of advanced cataract surgery techniques, such as phacoemulsification, and improved access to surgical care, potentially influencing population-level trends. The cohort effect curve (Figure 4D) demonstrated a non-linear pattern. The rate ratios declined among cohorts born before 1930, followed by a moderate increase between the 1930 and 1940 birth cohorts, and subsequently reached a plateau after 1950. This trend suggests that generational differences in cataract risk were most pronounced among earlier cohorts, while those born after 1950 experienced relatively stable risk levels. The observed variations may reflect historical transitions in early-life exposures, nutrition, education, and access to basic healthcare, which began to stabilize for post-1950 cohorts as public health infrastructure improved globally.

Together, these APC analyses underscore the compound influences of age-related biological risk, period-specific health system improvements, and generational differences shaped by historical conditions in determining the global burden of cataract among older adults.

### Relationship between cataract prevalence and socio-demographic development at global, regional, and national levels

**Figure 5:**
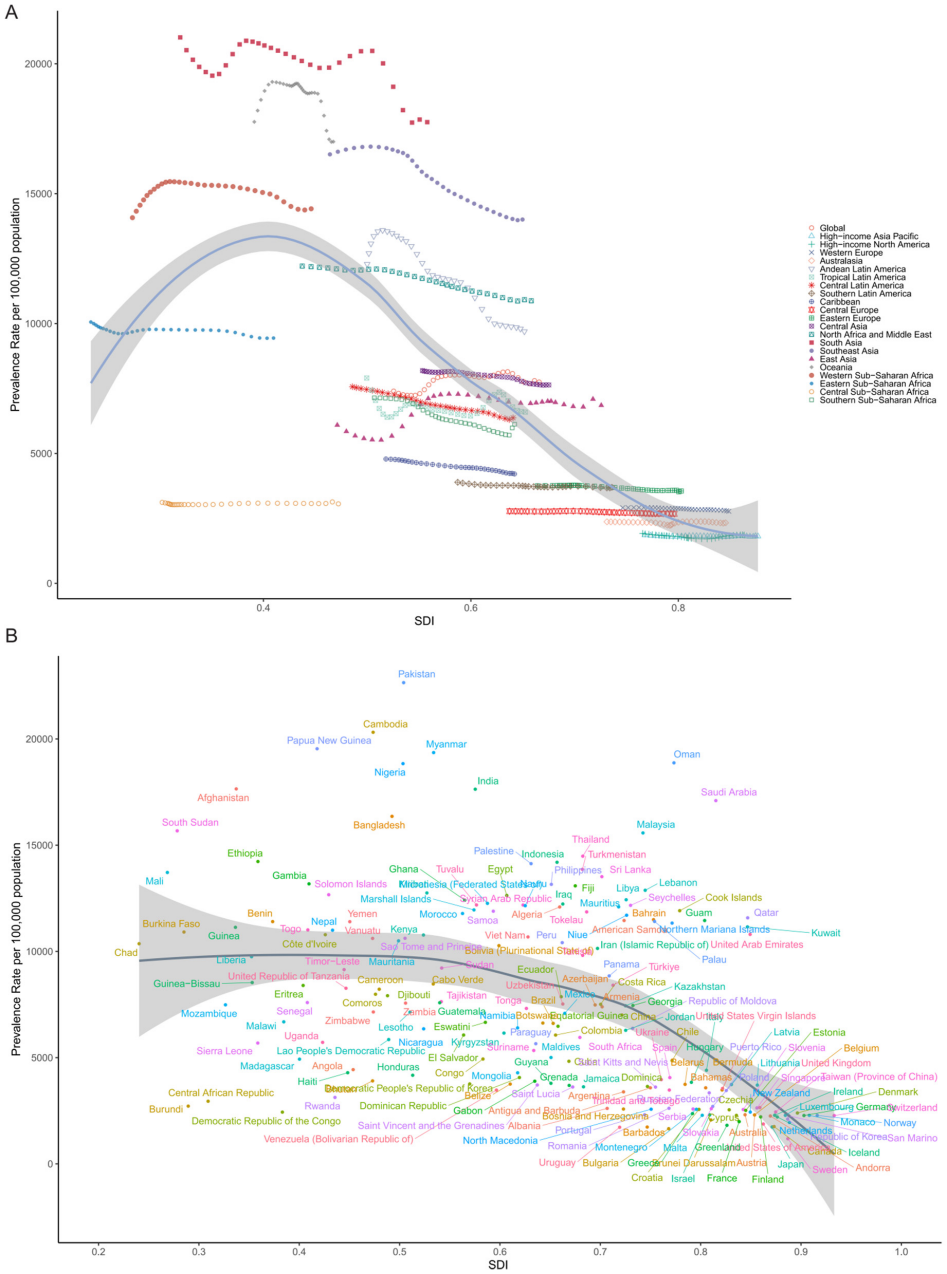
Association between SDI and ASPR of cataract in 2021. **(A)** Across 21 GBD regions, each point represents one region, with SDI on the *X*-axis and ASPR (per 100,000 population) on the *Y*-axis. A non-linear LOESS-smoothed curve illustrates the overall trend. **(B)** Across 204 countries and territories, each point represents one country.

The global association between ASPR of cataract and the SDI followed a distinct non-linear pattern, as illustrated in Figure 5A. ASPR remained relatively low in countries with very low SDI (< 0.3), increased steadily as SDI rose, and peaked around SDI = 0.4. Beyond this threshold, prevalence declined progressively. This inverted U-shaped relationship suggests that cataract burden does not increase linearly with development and may reflect the combined effects of underdiagnosis and limited treatment availability at low SDI levels, increased detection with rising access in mid-SDI settings, and enhanced surgical intervention and prevention in high-SDI contexts.

At the regional level, many trajectories remained relatively flat over time, indicating that increasing SDI did not always correspond with reductions in cataract burden. In Southeast Asia, Sub-Saharan Africa, and parts of South Asia, for example, ASPR remained high despite gradual improvements in socio-demographic indicators. This lack of convergence suggests that economic growth and demographic transition alone are insufficient, and that investments in public eye health infrastructure, human resources, and surgical access remain critical bottlenecks.

More striking differences emerged at the national level. Figure 5B illustrates substantial variation in ASPR among countries with comparable SDI. At SDI ≈ 0.6, China reported a considerably lower ASPR than the Philippines, Indonesia, or Myanmar, despite occupying a similar development band. This discrepancy may reflect differences in cataract surgical coverage (CSC), national eye health priorities, public insurance coverage for ophthalmic services, and the success of long-term blindness prevention programs, such as China's National Plan for the Prevention and Treatment of Blindness.

Conversely, countries such as India, Bangladesh, and Nigeria, though advancing socio-economically, remained in the upper segment of the curve with high ASPRs, possibly reflecting a large backlog of untreated cataract cases and regional disparities in health service delivery. These findings highlight that ASPR is not solely determined by socio-demographic development, but also by how well countries translate growth into functional, equitable, and accessible ophthalmic care systems. The observed heterogeneity underscores the need for targeted strategies that address local health system gaps, especially in regions where cataract burden remains disproportionately high despite developmental gains.

### Decomposition analysis of changes in cataract prevalence by aging, population growth, and epidemiological change

**Figure 6:**
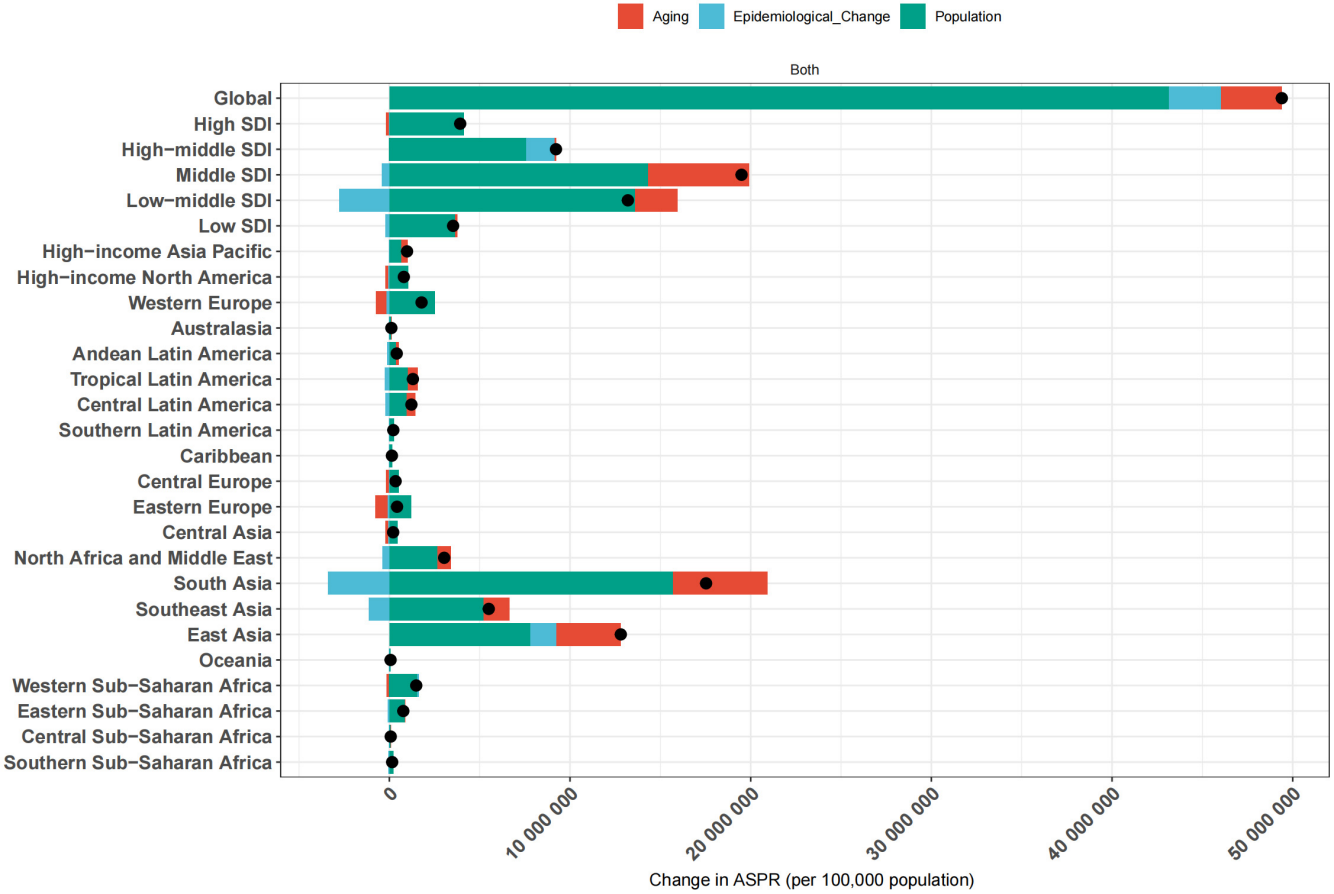
Decomposition of ASPR changes of ocular tumors by global, SDI, and regional levels from 1990 to 2021. The bar plots illustrate the contributions of three major components-population growth, population aging, and epidemiological change-to the net change in ASPR of ocular tumors over the study period. Results are presented for the global average, five SDI quintiles (low, low-middle, middle, high-middle, and high), and 21 GBD regions. Each bar represents the total ASPR change, with individual segments color-coded to denote the specific contribution of each component.

Decomposition analysis was performed to evaluate the relative contributions of population aging, population growth, and epidemiological change to the change in age-standardized prevalence of cataract across global, SDI, and regional levels (Figure 6). Epidemiological change denotes the variation in age-specific prevalence rates over time due to shifts in disease risk, diagnosis, or treatment-after accounting for changes in population size and age structure. At the global level, the total increase in cataract cases was largely attributable to population growth (87.4%), with smaller contributions from aging (6.8%) and epidemiological change (5.8%). This indicates that demographic expansion remains the dominant force, but changes in disease risk also play a measurable role. Across SDI strata, distinct patterns emerged. In low and low-middle SDI regions, the overall increase was primarily driven by population growth and aging. However, both regions showed negative epidemiological contributions, indicating that improvements in disease risk somewhat mitigated the burden. In contrast, high-middle SDI was the only group where all three components were positive, including epidemiological worsening, suggesting that disease burden is rising despite moderate development, potentially due to service delivery gaps. In high SDI regions, aging and epidemiological change both had negative impacts, counteracting the effect of population growth, resulting in a modest net increase. This reflects mature health systems with improved surgical coverage and disease control.

At the regional level, the largest absolute increase was observed in South Asia, the total number of cataract cases increased by 17.53 million from 1990 to 2021, with population growth contributing 15.67 million (89.4%), population aging 5.25 million (29.9%), and epidemiological change reducing the burden by 3.39 million (−19.3%). In contrast, Western Europe, High-income Asia Pacific, and High-income North America experienced epidemiological improvements, with net gains in total cases being primarily due to population growth, while aging and risk-adjusted prevalence contributed to reductions.

These findings underscore that while population growth and aging remain the principal drivers of cataract burden worldwide, the role of epidemiological change varies considerably. In some settings, improved eye care services have mitigated demographic pressures, while in others, service gaps and delayed access have allowed prevalence to rise. This decomposition highlights the critical need for aligning healthcare system capacity with demographic realities to reduce the growing burden of cataract.

### Global inequality in cataract burden: absolute and relative perspectives

To comprehensively assess global inequality in cataract burden, we analyzed both absolute and relative disparities using the SII and CII, respectively, across the years 1990 and 2021.

**Figure 7:**
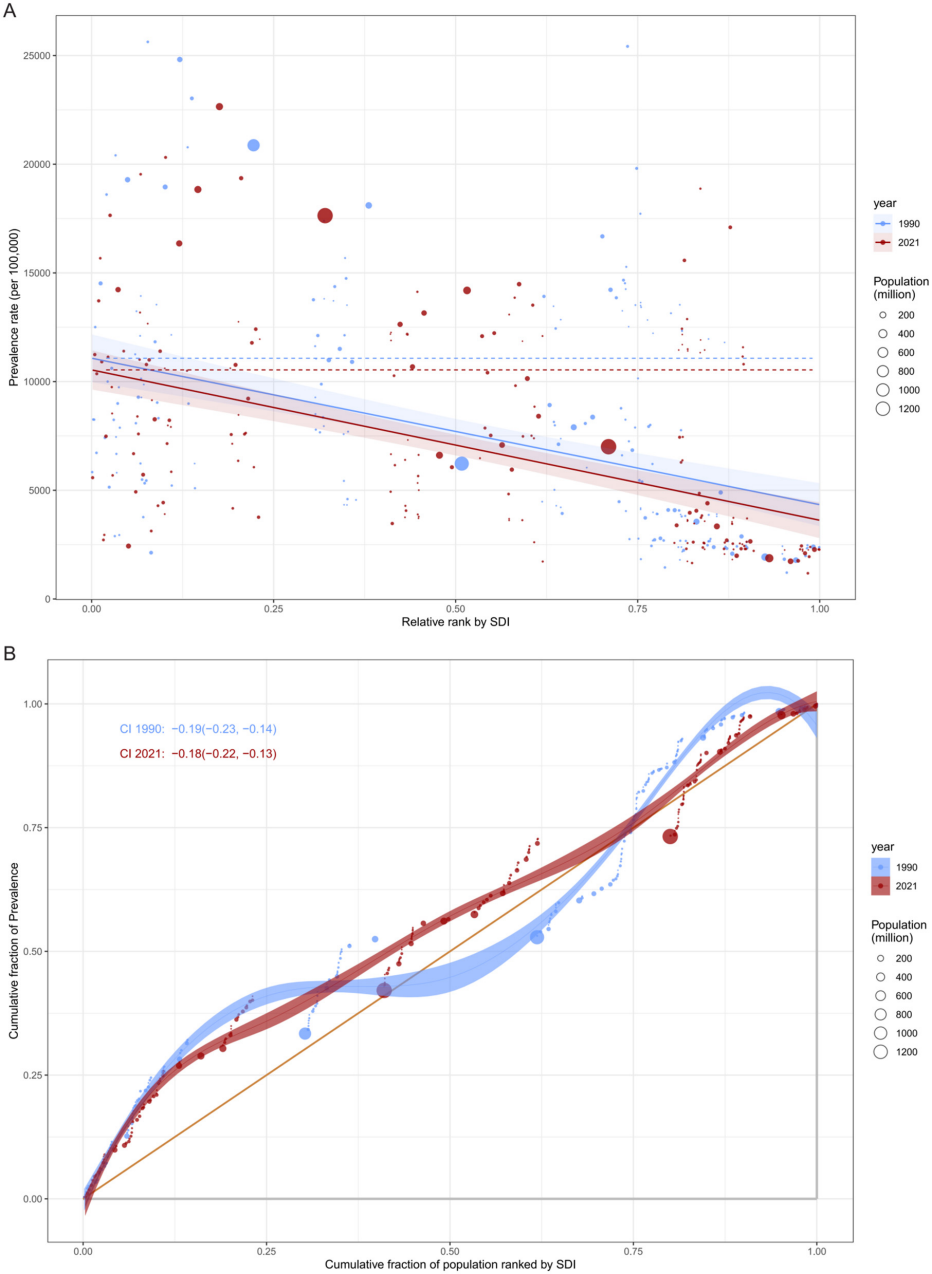
Global inequality in ASPR of cataract by SDI in 1990 and 2021. **(A)** Slope Index of Inequality (SII) illustrating the absolute inequality in cataract prevalence associated with SDI. The *x*-axis denotes the relative rank of populations by SDI (from lowest to highest), and the *y*-axis indicates prevalence rate per 100,000 population. Fitted lines represent inequality slopes in 1990 and 2021. **(B)** Concentration Index (CI) curves illustrating relative inequality in cataract prevalence. The *x*-axis shows the cumulative share of the population ranked by SDI, and the *y*-axis indicates the cumulative share of prevalence. Curves below the line of equality and negative CI values (CI < 0) signify a disproportionate burden in lower-SDI populations.

From the perspective of absolute inequality, health inequality regression curves (Figure 7) were constructed by plotting the ASPRs against the SDI for 204 countries. In both 1990 and 2021, the regression lines exhibited a clear negative slope, indicating a higher cataract burden in countries with lower SDI levels. The estimated slope coefficient was −6,735.83 in 1990 and −6,914.81 in 2021, demonstrating a persistently steep gradient in health burden across the development spectrum. Notably, the burden gap between the most and least developed countries increased from 6,735.8 per 100,000 in 1990 to 6,914.8 per 100,000 in 2021. This suggests a widening absolute disparity in cataract prevalence, although the slope change was modest.

In terms of relative inequality, concentration curves and corresponding CII values further quantified the distribution of cataract burden across socioeconomic strata. Both in 1990 and 2021, the concentration curves lay below the line of equality, indicating a “pro-poor” distribution-that is, the cataract burden was disproportionately concentrated among lower-SDI countries. The calculated CII decreased only slightly over time, from −0.185 (95% CI: −0.230 to −0.138) in 1990 to −0.179 (95% CI: −0.225 to −0.134) in 2021, with overlapping confidence intervals suggesting no statistically significant change.

Together, these findings highlight the persistent and multidimensional nature of global inequality in cataract burden. While marginal improvements may reflect global health initiatives and cataract programs in select regions, the overall picture remains one of substantial inequality, especially affecting less developed nations. Reducing these disparities will require targeted investments in cataract surgical services, integrated aging care, and the strengthening of national health systems in low- and middle-income countries.

### Frontier analysis of age-standardized prevalence and sociodemographic development

The frontier analysis revealed substantial heterogeneity in cataract burden relative to development levels across countries from 1990 to 2021. Despite overall improvements, many countries remained markedly above the theoretical minimum burden achievable at their respective SDI levels.

**Figure 8:**
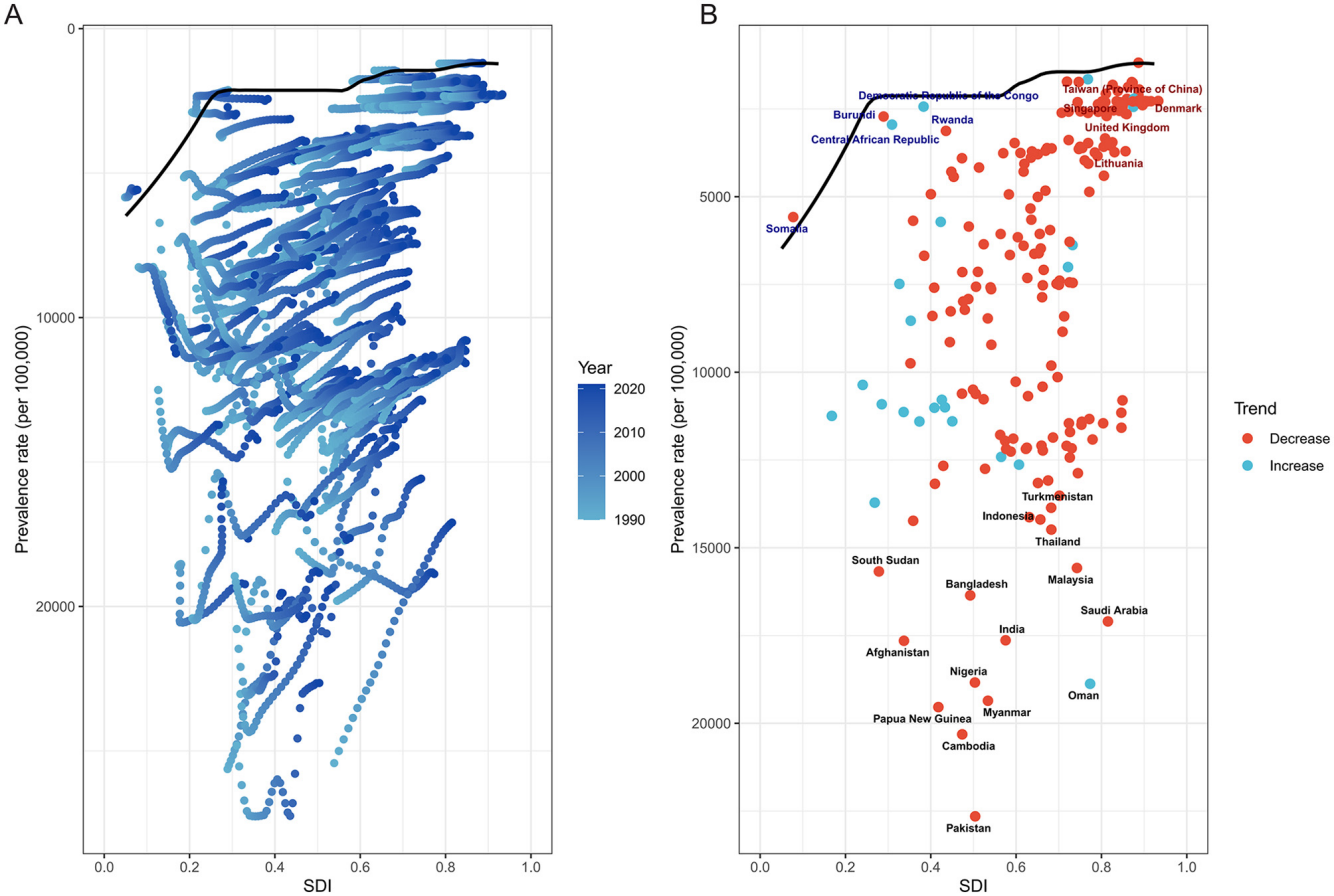
Frontier analysis of ASPR of ocular tumors by SDI from 1990 to 2021. **(A)** Scatterplot shows temporal evolution of each country's ASPR relative to SDI, with LOESS-derived frontier line representing optimal performance. Many countries lagged behind the frontier, indicating underperformance relative to their developmental level. **(B)** Highlighted are the 15 countries with the greatest distance from the frontier (black text), the five low-SDI countries (<0.50) closest to the frontier (blue text), and the five high-SDI countries (>0.85) farthest from the frontier (red text). Red-colored dots indicate declining burden over time, while blue-colored dots represent increasing trends. Large efficiency gaps persist across multiple regions, underscoring unrealized health potential.

In Figure 8A, while a general downward shift in prevalence was observed over time in most countries, many low- and middle-SDI countries persistently clustered above the efficiency frontier, indicating underutilized health potential. Only a minority of countries approached the frontier line, suggesting optimal alignment between development status and cataract burden control. Notably, several countries, particularly in sub-Saharan Africa and South Asia, remained far from the frontier despite modest SDI gains, reflecting constrained progress in disease prevention and treatment accessibility.

Figure 8B highlights the top 15 countries with the greatest absolute deviation from the frontier. These nations-regardless of SDI level-demonstrated significant inefficiencies in cataract burden reduction. In contrast, five low-SDI countries, including Rwanda and the Central African Republic, achieved burden levels close to the frontier, suggesting comparatively effective cataract control given limited development. Among high-SDI countries, five exhibited prominent underperformance, such as Lithuania, where relatively high prevalence persisted despite advanced sociodemographic conditions.

The trajectory of burden change also varied considerably. Several countries with large performance gaps nonetheless demonstrated substantial reductions in prevalence over the study period (e.g., India, Nigeria), while others with relatively low absolute burden exhibited increasing trends, underscoring emerging challenges (e.g., Central African Republic). These patterns point to divergent health system efficiencies and underscore the importance of not only improving access but also ensuring effective implementation of cataract prevention and surgical programs.

Collectively, this analysis reveals that narrowing the gap between observed and frontier-level prevalence remains a global challenge. Addressing this unrealized health potential will require tailored interventions that account for local healthcare infrastructure, economic constraints, and demographic shifts.

## Discussion

This study offers a thorough evaluation of the global, regional, and national burden of cataract among individuals aged 60 years and older, with a specific emphasis on ASPR in 2021. The findings indicate that cataract continues to be a significant global health issue within the aging population, characterized by considerable geographic variability. In 2021, the global ASPR among older adults was 7,748.5 per 100,000 population, with notably higher rates observed in regions such as South Asia, Oceania, and Sub-Saharan Africa, while significantly lower rates were documented in high-income regions, including Western Europe and the High-income Asia Pacific. This pronounced regional disparity highlights the impact of health system capacity, access to surgical services, and socioeconomic development on the burden of cataract.

A notable pattern observed was the concentration of high cataract prevalence in countries with low and lower-middle SDI. These regions not only experienced the highest disease burden but also demonstrated an inverted U-shaped relationship between ASPR and SDI. This pattern likely indicates a “double burden” scenario, where increased diagnostic capacity resulting from epidemiological transitions coexists with persistent deficiencies in surgical infrastructure. In numerous mid-SDI countries, enhanced awareness and screening have led to increased detection of cataract cases; however, the limited availability and affordability of cataract surgery continue to impede the provision of definitive treatment (25, 26). This discrepancy between diagnosis and treatment coverage may partially account for the elevated prevalence observed in these settings.

Previous global assessments of cataract burden, including those based on earlier GBD datasets, have typically analyzed all-age populations and presented mainly descriptive trends in prevalence and surgical coverage (18–20, 22). These studies, while valuable, often lacked integrated analytical frameworks that could separate demographic pressures from changes in risk, quantify inequality across socio-demographic gradients, and benchmark health system performance relative to development level. In contrast, our study applies a multidimensional approach that focuses exclusively on adults aged ≥60 years, incorporates Age-Period-Cohort modeling, Das Gupta decomposition, and both absolute and relative inequality metrics, and uses SDI-ASPR frontier analysis to reveal efficiency gaps between countries with similar resources. Furthermore, by providing detailed sex-specific analyses across 5-year age intervals up to ≥95 years, we identify persistent female disadvantage and widening absolute inequality, which have not been systematically quantified in previous literature. This comprehensive and age-targeted framework yields policy-relevant insights that extend beyond the descriptive scope of earlier work and directly support equity-focused, backlog-reduction, and efficiency-improvement strategies.

Our findings underscore several underlying factors contributing to the persistent and heterogeneous prevalence of cataracts among the elderly. These factors are rooted in demographic transitions, health system performance, gender inequity, and the rate of technological adoption, all of which collectively influence the observed trends across countries and regions. Firstly, population aging and growth are fundamental drivers of the increasing cataract burden. Decomposition analysis indicates that over 90% of the global rise in cataract cases from 1990 to 2021 can be attributed to demographic expansion, highlighting the inevitable impact of an expanding elderly population. Furthermore, age-stratified analysis demonstrates a consistent increase in ASPR with advancing age, reaching a peak in individuals aged 95 years and older. This age-dependent escalation underscores the intrinsic association between cataract formation and biological aging. Factors such as oxidative stress accumulation, mitochondrial dysfunction, prolonged exposure to ultraviolet radiation, and systemic comorbidities (e.g., diabetes, hypertension) are well-documented contributors to age-related lens opacification (27–33). The disproportionate burden observed in the oldest age groups emphasizes the necessity of incorporating cataract prevention and treatment into comprehensive geriatric care strategies.

The readiness of health systems is crucial in determining whether the burden of cataract is effectively managed or allowed to accumulate unchecked. In numerous low- and middle-SDI countries, CSC remains inadequate, particularly among underserved populations (34). Despite heightened awareness and increased diagnostic uptake, the capacity to provide timely and affordable cataract surgery is often constrained by a shortage of trained ophthalmologists, insufficient infrastructure, and financial barriers. This service gap is a primary factor contributing to the elevated ASPR observed in these regions. In contrast, in high-SDI countries, the decreasing prevalence of cataracts can largely be attributed to the widespread adoption of advanced surgical technologies, such as phacoemulsification, alongside well-structured national screening programs and integrated elderly care services (35). These systems not only enhance access to surgical treatment but also reduce delays in intervention, thereby minimizing the accumulation of disease burden within the population. Our frontier analysis shows divergent prevalence trends between countries of similar SDI levels. This suggests that public health outcomes depend not only on economic development but also on effectively translating resources into services.

The persistently elevated cataract burden observed among older women can be attributed to both biological and sociocultural determinants (36). Firstly, hormonal changes, particularly the decline in estrogen after menopause, may reduce lens antioxidant defenses and increase susceptibility to oxidative damage (37, 38). While women's longer life expectancy partially accounts for their increased exposure to age-related lens opacification, it does not fully explain the extent of the disparity. In numerous regions, particularly those with low SDI levels, women encounter significant obstacles in accessing eye care services (17, 39). These obstacles include limited decision-making autonomy, economic dependency, and restricted mobility. Such structural barriers diminish the likelihood of receiving timely surgical treatment, resulting in a higher prevalence of untreated cases among elderly women (40). Furthermore, the absence of targeted policy interventions to address these gender-based disparities has perpetuated their persistence over time. Our analysis indicates that despite moderate advancements in surgical technology and healthcare access on a global scale, the relative inequality in cataract burden has remained largely unchanged since 1990. This finding suggests that general improvements in health systems may be insufficient to bridge gender gaps unless they are accompanied by equity-focused strategies.

Temporal trends in cataract prevalence were also shaped by health technology adoption and public health policy. The observed slowing or reversal of period effects after 2005 coincided with the global expansion of cataract surgery programs and technological shifts toward minimally invasive, high-throughput procedures. These innovations not only improved surgical outcomes but also reduced procedural time and costs, allowing for greater volume and access (41, 42). Nevertheless, such benefits have not been equitably distributed. Many countries continue to lag behind in the adoption of modern techniques due to resource constraints and weak governance. The frontier analysis further highlighted national-level efficiency gaps, where some countries performed substantially worse than expected given their level of development. This inefficiency reflects systemic barriers beyond income or demographic characteristics-including underinvestment in human resources, poor service coordination, and weak accountability structures. Bridging this implementation gap is crucial to closing the cataract burden divide. Countries that deviate substantially above the frontier, such as India and Nigeria, may prioritize backlog-reduction strategies and CSC expansion. In high-SDI settings such as Lithuania, frontier deviations highlight systemic inefficiencies that require targeted policy reforms in human resources and service coordination.

The findings of this study underscore the urgent necessity for region-specific, equity-focused strategies to mitigate the global cataract burden among the elderly. In countries with high prevalence rates, particularly in South Asia and Sub-Saharan Africa, it is imperative to enhance CSC through subsidized or free national surgery programs and decentralized service delivery to effectively address the treatment gap. Successful national initiatives demonstrate the feasibility and impact of such strategies. For example, China's National Plan for the Prevention and Treatment of Blindness expanded cataract surgical coverage in rural areas through government subsidies and infrastructure investment, substantially reducing cataract blindness and improving equity of access. Similarly, India's National Programme for Control of Blindness and Visual Impairment has implemented large-scale backlog-reduction campaigns, mobile outreach services, and public–private partnerships, which significantly increased surgical output and lowered the burden of untreated cataract. For countries with low SDI, the establishment of community-based vision screening programs, particularly targeting older adults and high-risk groups such as women, is crucial for improving early detection and reducing access barriers. In regions with middle SDI, where diagnostic capacity has surpassed treatment provision, policymakers should prioritize efficiency-driven “catch-up” strategies, including backlog-reduction campaigns and the integration of cataract services into health insurance schemes. Across all contexts, investment in cataract registries, real-time monitoring systems, and long-term forecasting models is essential for informed planning. These combined efforts are critical to bridging the cataract care gap and ensuring healthy vision for aging populations globally.

This study possesses several limitations that merit careful consideration. Firstly, the estimates used in this study were derived from the GBD 2021 framework, which applies statistical modeling to synthesize data from heterogeneous sources. In countries with limited, outdated, or poor-quality primary data, this reliance on model-based extrapolation may lead to increased uncertainty, potentially affecting the precision of cross-country comparisons and inequality assessments (43). Secondly, our analysis focused on the overall age-standardized prevalence of cataracts without distinguishing structural subtypes (e.g., nuclear, cortical, posterior subcapsular) or etiologic categories (e.g., metabolic, drug-induced, secondary), which may differ in risk profiles and treatment outcomes (44, 45). The GBD 2021 cause list aggregates cataract and does not provide subtype-specific estimates due to limited and inconsistent data across countries. As such, we could not assess heterogeneity in trends or drivers by subtype. Thirdly, the study did not evaluate post-surgical outcomes or vision restoration rates, which are essential indicators of the true impact and effectiveness of cataract intervention programs. Lastly, the APC analysis assumes additive and independent contributions of age, period, and cohort effects, and is subject to the identifiability problem arising from their exact linear dependency. While we applied the estimable functions approach to mitigate this issue, the results should be interpreted as reflecting relative patterns and temporal turning points rather than definitive quantitative separations of each effect. These assumptions may influence the precision of individual effect estimates, although the overall trends identified are consistent with established epidemiologic patterns of cataract. Future research should incorporate real-world, individual-level data to validate and refine burden estimates, assess disparities in surgical outcomes, and explore interactions between cataracts and other chronic conditions such as diabetes and hypertension. Integrating such multifaceted data will facilitate more precise targeting of interventions and promote a comprehensive approach to vision and aging health.

## Conclusion

This study provides the most current and comprehensive global analysis of the cataract burden among the elderly, utilizing GBD 2021 data to elucidate long-term trends, regional disparities, and underlying systemic factors. It identifies significant deficiencies in surgical coverage, gender disparities, and the efficiency of care delivery, particularly in low- and middle-SDI contexts. These findings offer crucial evidence to guide national eye health planning, resource allocation, and the development of global strategies for promoting healthy aging. As the global population continues to age, addressing the cataract burden will be essential for preventing avoidable visual impairment and achieving universal eye health equity, in alignment with the goals of the WHO Universal Eye Health Plan and the broader global agenda for vision health.

## Data Availability

The original contributions presented in the study are included in the article/Supplementary material, further inquiries can be directed to the corresponding authors.

## Supplementary material

Supplementary Figure S1 Projected global burden of cataract among adults aged ≥60 years to 2050. **(A)** Projected absolute number of prevalent cases. **(B)** Projected ASPRs per 100,000 population.
