# Supplementary figures and images for: Global, regional, and national burden of cataract among older adults from 1990 to 2021: a comprehensive analysis based on the global burden of disease study 2021
Source: Front Med (Lausanne). 2025 Sep 4;12:1679828. doi: 10.3389/fmed.2025.1679828 (PMC12443760; doi:10.3389/fmed.2025.1679828)

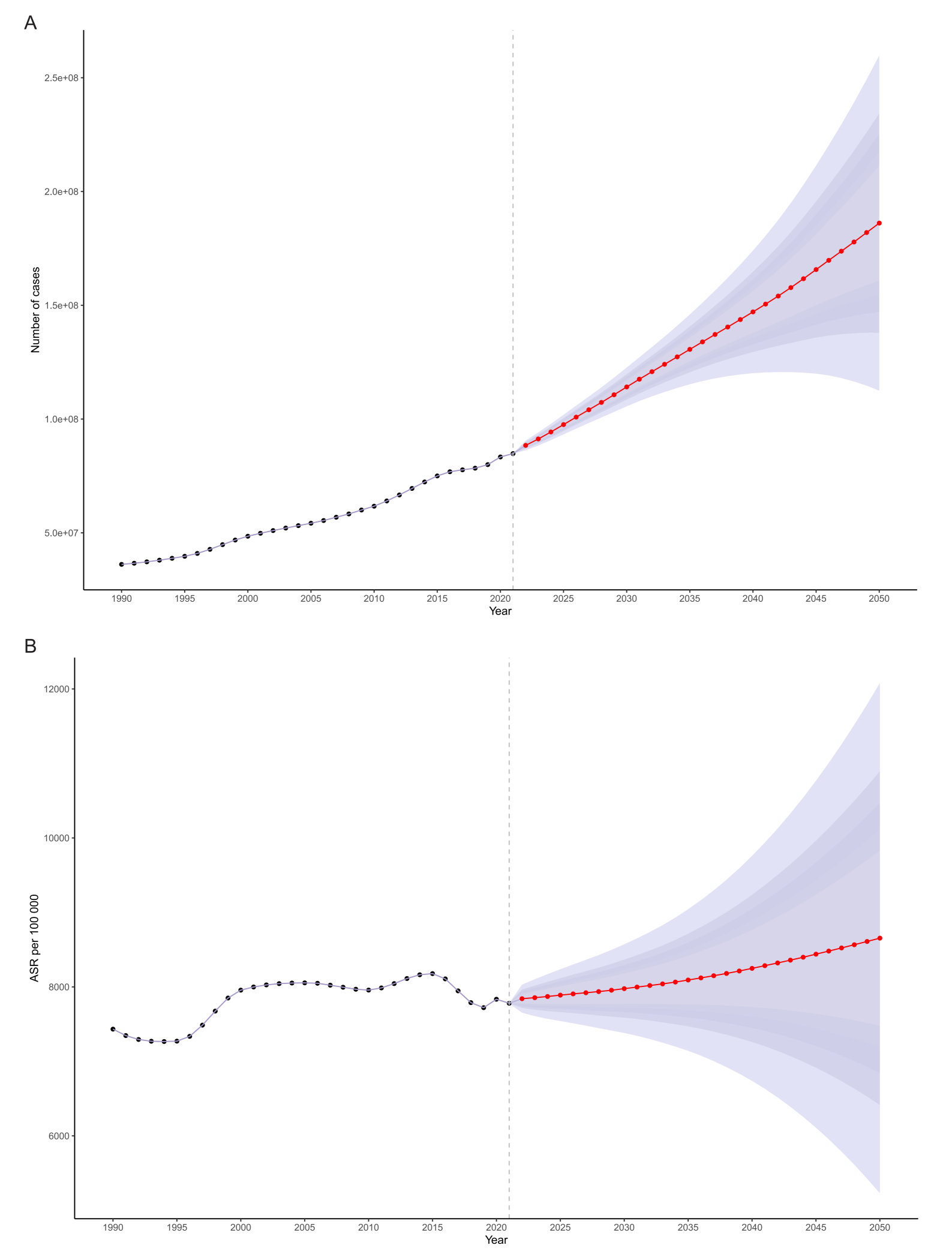

Supplement: Supplementary Figure S1 — Projected global burden of cataract among adults aged ≥60 years to 2050. (A) Projected absolute number of prevalent cases. (B) Projected ASPRs per 100,000 population. [file Image_1.tiff]
